# The Association Between Peripheral Complete Blood Count Parameters and Depressive Symptoms: A Cross-Sectional Study in Eastern Sudan

**DOI:** 10.3390/jcm15176909

**Published:** 2026-09-07

**Authors:** Sami S. Alharthi, Saeed M. Omar, Hatim Y. Alharbi, Afnan A. Alwabili, Ishag Adam

**Affiliations:** 1 Department of Medicine, College of Medicine, Taif University, Taif 21944, Saudi Arabia; s.s.alharthi@tu.edu.sa; 2 Faculty of Medicine and Health Sciences, University of Gadarif, Gadarif 32211, Sudan; drsaeedomar@gaduniv.edu.sa; 3 Department of Psychiatry, College of Medicine, Qassim University, Buraydah 52389, Saudi Arabia; hy.alharbi@qu.edu.sa; 4 Department of Obstetrics and Gynecology, College of Medicine, Qassim University, Buraydah 52389, Saudi Arabia; ia.ahmed@qu.edu.sa

**Keywords:** depression, complete blood count, hematocrit, hemoglobin, mean platelet volume, age, Sudan

## Abstract

**Background:** Depression is a leading global cause of disability, increasingly recognized as a systemic condition involving low-grade immune-inflammatory activation and neuroendocrine dysregulation. Although complete blood count (CBC) parameters provide an accessible window into systemic physiology, empirical data from African countries, including Sudan, remain scarce. This study evaluated the associations between peripheral CBC parameters and depressive symptoms among adults in Eastern Sudan. **Methods:** A multistage community-based survey was conducted in Gadarif in Eastern Sudan. The sociodemographic characteristics and clinical data were collected through a face-to-face interview. Depressive symptoms were assessed through the Patient Health Questionnaire-9. Adults with a PHQ-9 score ≥ 10 were considered to have depressive symptoms. CBC parameters were assessed via venous blood samples using automated hematology analyzers. Multivariable linear regression with continuous PHQ-9 scores was performed. **Results:** Three hundred and thirty-two adults were enrolled. Of the 332 subjects, 166 (50.0%) adults were males, and 102 (30.7%, 95% CI = 25.7–35.7%) had depressive symptoms. There was a significantly higher median platelet count (308.0 × 10^9^/L vs. 291.5 × 10^9^/L; *p* = 0.025) and plateletcrit count (0.3% vs. 0.2%; *p* = 0.028), and a significantly lower hematocrit count (37.7% vs. 38.6%; *p* = 0.043) in adults with depressive symptoms. In the adjusted multivariable linear regression models, erythrocyte oxygen-carrying parameters remained associated with PHQ-9 scores, including hematocrit (B = −0.355, 95% CI = −0.511–−0.199, *p* < 0.001) and hemoglobin (B = −0.621, 95% CI = −1.191–−0.051, *p* = 0.033). Conversely, thrombocyte activation markers were associated with PHQ-9 scores, including mean platelet volume (MPV; B = 1.131, 95% CI = 0.221–2.041, *p* = 0.015) and total platelet count (B = 0.012, 95% CI = 0.00001–0.024, *p* = 0.047). Leukocyte profiles showed no association with PHQ-9 scores. **Conclusions:** Peripheral complete blood count parameters, specifically lower hematocrit and hemoglobin, alongside elevated MPV and platelet count, were associated with depressive symptoms in Eastern Sudan. CBC panels may a be useful inflammatory marker tool to detect the correlations with PHQ-9 scores in resource-limited primary care settings.

## 1. Introduction

Depression is a major global health concern and one of the leading causes of disability worldwide, contributing significantly to the global burden of disease and impairing individual functional capacity [1]. Despite its high prevalence and socio-economic impact, the precise pathophysiological mechanisms underlying depression are not yet fully understood. Growing evidence suggests that depression is not merely a central nervous system disorder but may be a systemic condition associated with persistent low-grade immune-inflammatory activation, systemic oxidative stress, and altered microvascular dynamics [2,3].

Central to this physiological cross-talk is the systemic inflammatory response, which triggers secretion of pro-inflammatory cytokines/chemokines, e.g., tumor necrosis factor-alpha (TNF-alpha) and interleukin-6 (IL-6) [4]. These cytokines can alter peripheral blood cell dynamics and hematopoietic lineages in distinct ways. First, in terms of erythrocyte indices, chronic low-grade inflammation can disrupt iron metabolism via hepcidin activation, leading to blunted erythropoiesis and reduced iron availability. Consequently, reductions in hemoglobin and hematocrit may be associated with depressed mood, reduced oxygen-carrying capacity, and chronic neuro-fatigue [5,6]. Indeed, longitudinal evidence demonstrates that individuals with depressive symptoms may be at risk of developing anemia over time [7]. Moreover, it has been shown that when bulk peripheral mean hemoglobin levels fall within normal diagnostic thresholds, individuals with depressive symptoms had subtle subclinical hemoglobin deficits in smaller red cell fractions [8]. Second, in terms of platelet dynamics, serotonergic pathways play a dual role in mood regulation and platelet function. Platelets share striking biochemical similarities with central serotonergic neurons, storing most peripheral serotonin in dense granules [9]. Immune activation and altered serotonin transport can induce platelet hyperreactivity [10], which is reflected in shifts in platelet count and the platelets’ morphological indices, namely mean platelet volume (MPV) and platelet distribution width (PDW) [11,12]. Furthermore, recent investigation into altered immune states confirms that elevated MPV and altered PDW significantly correlate with depression severity and affective symptoms [13]. Clinical evaluation of platelet parameters in depressed cohorts further indicates that MPV significantly increases in tandem with depression severity and specific symptom clusters (such as somatization and hopelessness), highlighting the direct link between systemic inflammatory activation, altered platelet indices, and disease progression [12]. Large-scale evaluations in psychiatric cohorts further demonstrate that platelet numbers and morphological indices, including MPV, exhibit distinct alterations across major affective disorders, confirming characteristic platelet reactivity patterns in unipolar depression [14].

Third, in terms of leukocyte responses, glucocorticoid secretion results from the hypothalamic–pituitary–adrenal (HPA) axis and can directly drive shifts in white blood cell (WBC) subpopulations and immune activation during systemic stress [15,16]. Furthermore, severe stress and immune activation trigger profound neuroendocrine feedback loops that reconfigure peripheral immune cell populations [17]. Clinical investigations in drug-naive depressed cohorts further confirm that systemic immune activation significantly elevates WBC counts and shifts leukocyte percentages alongside depressive symptom severity, highlighting routine hematological parameters as accessible peripheral markers of affective pathology [18]. It has been suggested that treatment of resistant depression needs to be operationalized as a staged, longitudinal condition, supported by systematic assessment of symptom domains, integrating clinical staging as well as neurobiological markers [19].

Although automated complete blood count (CBC) panels provide an accessible, cost-effective window into these hematological and inflammatory alterations, most existing studies have been conducted in high-income Western countries or specialist psychiatric facilities. It has been shown that routine laboratory findings are feasible for monitoring in everyday mental health practice [20]. Evidence from regional cohort studies, such as the Rafsanjan Youth Cohort Study in Iran, shows that associations between depressive disorder and specific indices (such as hematocrit, MPV, RDW, and leukocyte dynamics) are often sex-specific and influenced by population demographics [21].

While lower hematocrit is frequently attributed to inflammatory-mediated erythropoietic suppression, alternative etiologies common to Africa—namely iron deficiency, infectious exposure, and nutritional variation—must first be considered [22]. In sub-Saharan Africa, particularly in Sudan, data on peripheral hematological markers in adult populations with depression remain scarce. Recent epidemiological evidence highlights a high burden of mental distress in the region, with over one in five adults (21.7%) exhibiting depressive symptoms, a rate significantly magnified by ongoing armed conflict and internal displacement [23]. Furthermore, while previous research has often analyzed depression as a crude binary status, assessing depressive symptoms on a continuous scale provides greater clinical granularity into how peripheral biological markers shift alongside disease progression [24]. Indeed, CBC parameters function as continuous biological indices that may be a valuable tool to assess depressive symptom severity scores [25]. Local empirical evidence from Sudan corroborates this link: an evaluation of hematological profiles among depressed patients in Omdurman revealed a statistically significant inverse relationship between depression severity and hemoglobin levels (*p* = 0.007), despite overall mean complete blood count indices remaining within standard biological reference ranges [26]. These findings indicate that subclinical variations in hemoglobin, rather than overt clinical iron deficiency anemia alone, may serve as key hematological indicators of depressive symptom burden in the Sudanese population. Nationwide analyses further corroborate these findings, demonstrating that depressed individuals consistently exhibit significantly lower hematocrit levels and reduced red blood cell (RBC) counts [27]. Therefore, this study aimed to evaluate CBC parameters and their association with Patient Health Questionnaire-9 [PHQ-9] scores among adults in Gadarif, Eastern Sudan.

## 2. Materials and Methods

### 2.1. Study Design and Setting

This community-based, multistage stratified sampling survey was conducted among adults residing in Gadarif, Eastern Sudan, from March to June 2025. This study adhered strictly to the Strengthening the Reporting of Observational Studies in Epidemiology (STROBE) guidelines for cross-sectional studies [28].

### 2.2. Study Population and Eligibility Criteria

The target population comprised adults (aged ≥18 years) residing in Gadarif, including males and females. To prevent non-depressive hematological alterations from confounding the results, we excluded individuals with chronic active inflammatory or autoimmune diseases, hematological malignancies, acute systemic infections, or a history of blood transfusion within the preceding three months. We also excluded smokers and individuals receiving medications known to alter blood cell lineages (e.g., immunosuppressants, systemic corticosteroids, chemotherapy).

### 2.3. Sampling Strategy

A multistage random cluster sampling strategy was used to select participants across administrative units in Gadarif. For cluster selection, administrative units and residential neighborhoods were selected using probability proportional-to-size (PPS) sampling to obtain a representative sample of adults. Within each selected cluster, households were systematically sampled using a random walk procedure starting from a central administrative landmark. Every fifth household was visited, and eligible adults (≥18 years) were invited to participate. If there were no adults in the selected household or they refused to participate, the next household was chosen.

### 2.4. Data Collection and Clinical Measurements

Trained medical officers collected data through face-to-face interviews and physical examinations. A questionnaire was administered to collect data on age, sex, and smoking history.

Anthropometric Assessment: Height was measured to the nearest 0.1 cm using a portable stadiometer with participants standing barefoot. Weight was recorded to the nearest 0.1 kg using a calibrated digital scale with participants wearing light clothing. Body mass index (BMI) was calculated as weight in kilograms divided by height in meters squared (kg/m^2^) and categorized into underweight (<18.5 kg/m^2^), normal weight (18.5–24.9 kg/m^2^), overweight (25.0–29.9 kg/m^2^), and obese (≥30.0 kg/m^2^) [29].

Depressive Symptoms Assessment: The survey used the Arabic version of the Patient Health Questionnaire-9 (PHQ-9), which medical officers completed during face-to-face interviews. PHQ-9 is a nine-item screening instrument widely used to measure depressive symptoms [24]. The PHQ-9 measures responses to specific questions on a four-point Likert-type scale, with responses summed from 0 for “Not at all” to 3 for “Almost every day.” Total scores range from 0 to 27. Thus, higher PHQ-9 scores indicate higher levels of depressive symptoms and vice versa [24]. The PHQ-9 has shown adequate validity for screening depression and satisfactory internal consistency across a variety of populations [30,31]. PHQ-9 has been widely used in Sudan [32,33]. Based on previous studies, adults are considered to have depressive symptoms if PHQ-9 ≥ 10 [30,31].

### 2.5. Laboratory Analysis

After the physical assessment, we collected a 3 mL venous blood sample from each participant using aseptic venipuncture into ethylenediaminetetraacetic acid (EDTA) tubes. Blood samples were placed in insulated cold storage boxes (2–8 °C) and transported to the central laboratory, where automated CBC processing was performed within 2 h of collection. Parameters evaluated included the following: Erythrocyte Indices: hemoglobin, red blood cell count (RBC), hematocrit, mean corpuscular volume (MCV), mean corpuscular hemoglobin (MCH), mean corpuscular hemoglobin concentration (MCHC, g/dL), and red cell distribution width (RDW-CV, %). Leukocyte Profile: total white blood cell count (WBC), lymphocyte count, and granulocyte count. Platelet Indices: platelet count, mean platelet volume (MPV), platelet distribution width (PDW), and plateletcrit.

### 2.6. Measures to Control Bias

Several measures were taken to control for bias. Selection bias was controlled via multistage random cluster sampling. Information and measurement bias was standardized through the use of validated diagnostic instruments (PHQ-9), with direct anthropometric measurement rather than self-report, and blinding laboratory personnel analyzing CBC samples to participants’ clinical depression status. Preanalytical bias was minimized by enforcing strict blood collection, transport, and rapid processing protocols within 2 h.

### 2.7. Sample Size Calculation

The Sample Size Calculator for designing clinical research was used [34] following the previous guidelines for assessing correlation [35,36]. A sample size of 332 adults was calculated, assuming a minimum significant correlation (r = 0.15) between PHQ-9 score and other independent hematological parameters (e.g., hemoglobin, MCHC, and platelet parameters). This sample size (332) was appropriate to detect alpha (α, Type I Error Rate), which was set to 0.05. This gives a 95% confidence level and beta (β), Type II Error Rate, or power (1 − β), which was set to 0.80 (80%).

### 2.8. Statistical Analysis

Statistical analysis was performed using IBM SPSS Statistics (Version 22.0). The normality of continuous variables was assessed using the Shapiro–Wilk test. Because continuous variables were non-normally distributed, we reported medians and interquartile ranges (IQRs). Between-group comparisons for adults with and without depressive symptoms were analyzed using the non-parametric Mann–Whitney U test. Categorical variables were presented as frequencies and percentages and compared between groups using the chi-square test. Univariable (unadjusted) linear regression analysis was performed with the continuous PHQ-9 score as the dependent variable and age, sex, BMI, and hematological parameters as independent variables. For the primary association analysis, the continuous PHQ-9 score was used as the dependent variable in linear regression. Associations between each CBC parameter and PHQ-9 score were first examined in separate unadjusted linear regression models. Because several CBC parameters are biologically and mathematically correlated, highly collinear indices were not interpreted as independent effects within the same model without prior assessment of multicollinearity. For adjusted analyses, prespecified demographic covariates, including age, sex, and BMI, were considered as potential confounders. Each selected CBC parameter was then evaluated in a separate adjusted model including the prespecified covariates. Regression coefficients, standard errors (SEs), 95% CIs, and *p*-values were reported. Multicollinearity was assessed using the variance inflation factor (VIF > 4), and regression assumptions were assessed. In this study, all statistical tests were two-tailed. Statistical significance was established at *p* < 0.05.

## 3. Results

### 3.1. Baseline Characteristics

Three hundred and thirty-two adults were enrolled. Of the 332 adults, 166 (50.0%) were males. The median (IQR) age and BMI were 42.0 (33.0–53.0) years and 26.2 (22.7–29.8) kg/m^2^, respectively. Of the 332 subjects, 102 (30.7%, 95% CI = 25.7–35.7%) had depressive symptoms. Baseline demographic characteristics were comparable between the two groups, with no statistically significant differences observed (Table 1). There was no significant differences in age, sex, and BMI between adults with and without depressive symptoms.

#### 3.1.1. Comparison of Hematological Profiles Between Adults with and Without Depressive Symptoms

Hematological profiles differed significantly between the two groups. Adults with depressive symptoms exhibited a significantly higher median (IQR) platelet count compared to adults without depressive symptoms (308.0 [270.0–370.0] × 10^9^/L vs. 291.5 [243.8–346.5] × 10^9^/L; *p* = 0.025). Similarly, median (IQR) plateletcrit was significantly higher in adults with depressive symptoms (0.3%, 0.2–0.3) than in adults without depressive symptoms (0.2%, 0.2–0.3; *p* = 0.028). Conversely, the median (IQR) of PDW was significantly lower in adults with depressive symptoms (15.3%, 15.0–15.5%) than in adults without depressive symptoms (15.4%, 15.2–15.6%; *p* = 0.017), whereas MPV did not differ between the two groups (*p* = 0.480).

Erythrocyte parameters showed no significant differences in hemoglobin levels (*p* = 0.253) or total RBC count (*p* = 0.171), although subtle red cell alterations were present between the two groups. Hematocrit values were significantly lower in adults with depressive symptoms compared to adults without depressive symptoms (37.7%, IQR: 34.4–40.6 vs. 38.6%, IQR: 36.2–42.0; *p* = 0.043). In contrast, MCHC was slightly higher among adults with depressive symptoms (34.1 g/dL, IQR: 33.4–34.7) than adults without depressive symptoms (33.9 g/dL, IQR: 33.2–34.3; *p* = 0.037). MCV, MCH, and RDW did not differ significantly between the two groups (*p* > 0.05 for all comparisons). Leukocyte indices, including total WBCs (*p* = 0.419), lymphocyte count (*p* = 0.432), and granulocyte count (*p* = 0.148), were not different between the two groups.

#### 3.1.2. Factors Associated with PHQ-9 Scores (Unadjusted Model)

There was no association between age and PHQ-9 scores (B = −0.029, SE = 0.0306, *p* = 0.342). Similarly, BMI showed no independent linear relationship with PHQ-9 scores (B = −0.118, SE = 0.0785, *p* = 0.132).

Among erythrocyte parameters, hematocrit demonstrated an inverse association with PHQ-9 scores (B = −0.358, SE = 0.0809, *p* < 0.001), indicating that lower hematocrit levels were associated with higher depression scale scores. Similarly, hemoglobin showed a statistically significant negative association (B = −0.651, SE = 0.2741, *p* = 0.018) with PHQ-9 scores, while RBC count displayed a trend toward an inverse association (B = −1.087, SE = 0.5995, *p* = 0.070) with PHQ-9 scores. Other red cell indices, including MCV (*p* = 0.820), MCH (*p* = 0.660), MCHC (*p* = 0.407), and RDW-CV (*p* = 0.146), were not associated with PHQ-9 scores.

In terms of platelet dynamics, platelet count was positively associated with PHQ-9 scores (B = 0.011, SE = 0.0057, *p* = 0.047). MPV also showed a significant positive association with PHQ-9 scores (B = 0.929, SE = 0.4665, *p* = 0.046). Conversely, PDW showed an inverse independent relationship with depression scores (B = −1.423, SE = 0.6946, *p* = 0.040). Plateletcrit did not remain statistically significant in the adjusted model (B = 11.601, SE = 7.0963, *p* = 0.102).

Regarding WBC parameters, total WBC count showed a marginal positive trend toward higher PHQ-9 scores (B = 0.451, SE = 0.2393, *p* = 0.059), whereas lymphocyte count (*p* = 0.827) and granulocytes (*p* = 0.338) were not significantly associated with PHQ-9 scores (Table 2).

#### 3.1.3. Independent Association of PHQ-9 Scores (Multivariable Adjusted Model)

To identify the key hematological indicators independently associated with PHQ-9 scores (PHQ-9 score), we constructed multivariable linear regression models (Table 3).

In the adjusted models, hematocrit remained significantly inversely associated with PHQ-9 scores (B = −0.355, 95 CI = −0.511–−0.199, *p* < 0.001), indicating that lower hematocrit levels were independently associated with PHQ-9 scores. Hemoglobin also remained significantly and negatively associated with PHQ-9 scores (B = −0.621, 95 CI = −1.191–−0.051, *p* = 0.033).

Among platelet indices, MPV demonstrated a statistically significant positive association with PHQ-9 scores (MPV; B = 1.131, 95% CI = 0.221–2.041, *p* = 0.015. Platelet count also showed a significant positive association with PHQ-9 scores (B = 0.012, 95 CI = 0.00001–0.024, *p* = 0.047). In contrast, PDW and plateletcrit were not associated with PHQ-9 scores following multivariable adjustment. R-square = 0.091.

## 4. Discussion

Understanding the systemic biological parameters of affective disorders is critical for refining clinical risk stratification, particularly in resource-constrained primary healthcare settings. This community-based cross-sectional study evaluated peripheral CBC indices and their association with depressive symptoms, measured continuously via the PHQ-9, among adults in Gadarif, Eastern Sudan.

The multivariable models demonstrate two findings. First, erythrocyte oxygen-carrying parameters, specifically hematocrit and total hemoglobin concentration, demonstrate an inverse association with PHQ-9 scores. Second, morphological and quantitative markers of thrombocyte reactivity, specifically MPV and total platelet count, exhibit a positive association with PHQ-9 scores. Furthermore, these biological shifts occurred while median hematological parameters remained well within standard physiological reference limits for the region, as recently established among healthy Sudanese adults [37]. This highlights that subclinical variation across continuous cell lines is associated with PHQ-9 scores, even within localized normative reference intervals.

In the adjusted model, declining hematocrit and hemoglobin levels were associated with PHQ-9 scores. These empirical patterns closely mirror regional clinical observations in Central Sudan by Hussein and Ahmed [26], who reported a significant inverse correlation between depression symptoms and hemoglobin levels (*p* = 0.007) despite overall CBC profiles remaining within normal diagnostic limits. Globally, our findings align with prospective cohort data from Liu et al. [7], who established that depressive symptoms were associated with progressive hemoglobin decline over time in a dose-dependent manner, as well as community observations by Vulser et al. [6] and Lee [27]. Biologically, this continuous erythrocyte suppression could be explained by the cytokine–hepcidin axis. Chronic low-grade immune activation, a hallmark of major depression, elevates circulating pro-inflammatory cytokines such as IL-6 and TNF-alpha [2,4]. Cytokine-driven hepatic induction of hepcidin downregulates ferroportin, sequestering iron within the reticuloendothelial system and blunting bone marrow erythropoiesis [3]. Central nervous system tissues are uniquely sensitive to subtle hypoxic stress; microvascular hypoperfusion and neuro-fatigue exacerbate prefrontal cortex dysfunction, worsening core affective symptoms such as psychomotor slowing, cognitive blunting, and anhedonia [5,8].

Paralleling these erythrocyte alterations, our investigation highlights that altered thrombocyte dynamics were associated with PHQ-9 scores. In multivariable models, elevated MPV and total platelet count were associated with higher PHQ-9 scores. Because platelets share key biochemical features with central serotonergic neurons that actively transport and store serotonin (5-HT) within dense granules, they may serve as an accessible peripheral mirror of central neurochemical dysregulation [9]. Sustained psychological stress and HPA axis hyperactivation induce a pro-thrombotic, low-grade inflammatory state [10,15]. Under systemic inflammatory pressure, bone marrow megakaryopoiesis is accelerated, releasing younger, larger platelets with higher MPV into circulation. These dense-granulated platelets express higher surface receptor density and exert greater pro-inflammatory reactivity [13]. Our findings substantiate previous clinical studies by [11,12], confirming that elevated MPV parallels disease progression and affective distress. In contrast to erythrocyte and platelet dynamics, leukocyte subpopulations displayed no association with PHQ-9 after multivariable adjustment. Although the total WBC count showed a marginal positive trend in unadjusted analyses, lymphocyte and granulocyte counts were not statistically significant. While severe psychiatric distress may trigger pronounced leukocytosis driven by glucocorticoid resistance and HPA axis hyperactivation [16,18], regional variability remains common. As demonstrated by Jamali et al. [21] in the Rafsanjan Youth Cohort, peripheral hematological manifestations of mood disorders are frequently sex- and population-specific. In Eastern Sudan, endemic infectious exposures, environmental stressors, and background nutritional variation may introduce baseline leukocyte variance, making erythrocyte and platelet morphological indices more sensitive peripheral markers of affective pathology than total WBC counts.

### Strengths and Limitations

This study possesses notable methodological strengths. The multistage random cluster sampling design. Furthermore, evaluating community-residing adults in Gadarif avoided the referral bias inherent in hospital-based studies, capturing a realistic spectrum of mild-to-severe depression. Assessing depression continuously via the PHQ-9 preserved statistical power and elucidated biological dose–response relationships. Finally, conducting this study in Eastern Sudan, a region underrepresented in biological psychiatry despite high mental distress magnified by armed conflict and displacement [23], provides essential baseline empirical data. However, some limitations warrant consideration. First, the observational cross-sectional design precludes inferring direct causal directionality regarding whether subclinical hematological shifts trigger depressive symptoms or vice versa. Second, the PHQ-9 was interviewer-administered, which would be a source of reporting bias. Moreover, the reliance on a single PHQ-9 cutoff for depressive symptoms may oversimplify the spectrum of depression severity and does not account for possible cultural differences in symptom reporting. Third, although the sample size calculation is described, the study may still be underpowered to detect smaller effect sizes or to perform subgroup analyses (e.g., by sex or age group). Fourth, resource constraints prevented the simultaneous measurement of direct serum inflammatory biomarkers such as high-sensitivity C-reactive protein, IL-6, or complete iron panels (serum ferritin, hepcidin). Third, although we enforced major medical exclusions and adjusted for demographic covariates, unmeasured subclinical micronutrient variations (e.g., vitamin B12 or folate status) could partially influence red cell indices. Iron status, endemic infection, hemoglobinopathies, and menstrual status were not measured. Likewise, cognitive morbidities or declining and their link to biomarkers were not assessed [38,39].

## 5. Conclusions and Recommendations

In conclusion, this community-based study in Eastern Sudan establishes that peripheral hematological parameters, specifically hematocrit, hemoglobin, MPV, and total platelet count, are associated with PHQ-9 score. Crucially, these continuous hematological shifts occur within standard physiological reference limits. Future research is needed to integrate serum cytokine profiles, hepcidin levels, and longitudinal follow-up to establish causal pathways and evaluate the direction of associations with PHQ-9 score.

## Figures and Tables

**Table 1:** Comparing baseline socio-demographic and hematological characteristics of adults with and without depressive symptoms in Sudan, 2025.

Variable		Adults with Depressive Symptoms (n = 102)	Adults Without Depressive Symptoms (n = 230)	p
		Median (interquartile range)		
Age, years		43.0 (34.0–50.0)	45.0 (33.0–55.0)	0.396
Body mass index, kg/m^2^		25.9 (22.0–29.0)	27.1 (23.5–30.7)	0.389
Hemoglobin (g/dL)		13.0 (12.3–14.1)	13.4 (12.4–14.5)	0.253
Red blood cell count (RBC, ×10^12^/L)		4.6 (4.3–4.9)	4.7 (4.4–5.0)	0.171
Hematocrit, %		37.7 (34.4–40.6)	38.6 (36.2–42.0)	0.043
Mean corpuscular volume, FL		84.0 (81.7–86.3)	83.9 (81.7–87.4)	0.822
Mean corpuscular hemoglobin, pg		28.5 (27.5–29.5)	28.6 (27.4–29.8)	0.703
Mean corpuscular hemoglobin concentration, g/dL		34.1 (33.4–34.7)	33.9 (33.2–34.3)	0.037
Red cell distribution width, %		14.3 (13.7–15.0)	14.2 (13.6–15.0)	0.282
Total white blood cell count, ×10^9^/L		6.0 (4.9–7.5)	5.8 (5.1–7.1)	0.419
Lymphocyte count, ×10^9^/L		2.4 (2.1–3.1)	2.6 (2.1–3.0)	0.432
Granulocytes, ×10^9^/L		3.3 (2.4–4.1)	2.8 (2.2–3.8)	0.148
Platelet count, ×10^9^/L		308.0 (270.0–370.0)	291.5 (243.8–346.5)	0.025
Mean platelet volume, FL		8.4 (8.0–8.8)	8.4 (7.9–8.8)	0.480
Platelet distribution width, %		15.3 (15.0–15.5)	15.4 (15.2–15.6)	0.017
Plateletcrit, %		0.3 (0.2–0.3)	0.2 (0.2–0.3)	0.028
		Number (percentage)		
Sex	Male	48 (47.0)	118 (51.3)	0.524
	Female	54(53.0)	112(48.7)	

**Table 2:** Unadjusted linear regression analysis of complete blood count parameters and sociodemographic variables on continuous depression severity scores in adults, Gadarif, Eastern Sudan.

Parameter	Coefficient	95% Confidence Interval		Standard Error	p
		Lower	Upper		
Age, years	−0.029	−0.09	0.03	0.0306	0.342
Body mass index, kg/m^2^	−0.118	−0.27	0.04	0.0785	0.132
Hemoglobin, g/dL	−0.651	−1.19	−0.11	0.2741	0.018
Red blood cell count, ×10^12^/L	−1.087	−2.26	0.09	0.5995	0.070
Hematocrit, %	−0.358	−0.52	−0.2	0.0809	<0.001
Mean corpuscular volume, FL	−0.015	−0.14	0.11	0.0648	0.820
Mean corpuscular hemoglobin, pg	−0.076	−0.41	0.26	0.1722	0.660
Mean corpuscular hemoglobin concentration, g/dL	0.187	−0.25	0.63	0.2250	0.407
Red cell distribution width, %	0.429	−0.15	1.01	0.2954	0.146
Total white blood cell count, ×10^9^/L	0.451	−0.02	0.92	0.2393	0.059
Lymphocyte count, ×10^9^/L	0.142	−1.14	1.42	0.6537	0.827
Granulocytes, ×10^9^/L	−0.031	−0.09	0.03	0.0319	0.338
Platelet count, ×10^9^/L	0.011	0	0.02	0.0057	0.047
Mean platelet volume, FL	0.929	0.01	1.84	0.4665	0.046
Platelet distribution width, %	−1.423	−2.78	−0.06	0.6946	0.040
Plateletcrit, %	11.601	−2.31	25.51	7.0963	0.102

**Table 3:** Adjusted multivariable linear regression analysis of factors associated with PHQ-9 scores in Gadarif, Eastern Sudan.

Parameter	Coefficient	95% Confidence Interval	p	Covariates	Variance Inflation Factor
Hemoglobin, g/dL	−0.621	−1.191–−0.051	0.033	Age, sex, BMI	1.04
Hematocrit, %	−0.355	−0.511–−0.199	<0.001	Age, sex, BMI	1.14
Platelet count, ×10^9^/L	0.012	0.00001–0.024	0.047	Age, sex, BMI	1.18
Mean platelet volume, FL	1.131	0.221–2.041	0.015	Age, sex, BMI	1.07
Platelet distribution width, %	−1.132	−2.528–0.264	0.112	Age, sex, BMI	1.03

BMI: body mass index.

## Data Availability

The data supporting the current study’s findings are available from the corresponding author upon reasonable request.

## Abbreviations

BMI: body mass index, CBC: complete blood count, HPA: hypothalamic–pituitary–adrenal, IL-6: interleukin-6, IQR: interquartile range, MCH: mean corpuscular hemoglobin, MCHC: mean corpuscular hemoglobin concentration, MCV: mean corpuscular volume, MPV: mean platelet volume, PDW: platelet distribution width, PHQ-9: Patient Health Questionnaire-9, RBC: red blood cell, RDW: red cell distribution width, RDW-CV: red cell distribution width–coefficient of variation, SD: standard deviation, SE: standard error, TNF-alpha: tumor necrosis factor-alpha, WBC: white blood cell.
